# Single, Double and ETL-Sandwiched PVPy Interlayer Effect on Charge Injection Balance and Performance of Inverted Quantum Dot Light-Emitting Diodes

**DOI:** 10.3390/polym15153308

**Published:** 2023-08-04

**Authors:** Collins Kiguye, Woo Jin Jeong, Gwang Hyun Jeong, Jin Ho Park, Hee Jung Kwak, Gun Woong Kim, Seok Hwan Jang, Jun Young Kim

**Affiliations:** Department of Semiconductor Engineering, Gyeongsang National University, 501 Jinjudaero, Jinju 52828, Gyeongnam, Republic of Korea; kiguye@gnu.ac.kr (C.K.); jeongwj95@gnu.ac.kr (W.J.J.); rhkddls3139@gnu.ac.kr (G.H.J.); wlsgh7470@gnu.ac.kr (J.H.P.); kwakhj@gnu.ac.kr (H.J.K.); kkw1584@naver.com (G.W.K.); alex2x@gnu.ac.kr (S.H.J.)

**Keywords:** maximum external quantum efficiency (EQE), turn-on voltage (V_on_), maximum luminance, current efficiency, electron transport layer (ETL), hole transport layer (HTL), reference, reference device

## Abstract

A desire to achieve optimal electron transport from the electron transport layer (ETL) towards the emissive layer (EML) is an important research factor for the realization of high performance quantum dot light-emitting diodes (QD-LEDs). In this paper, we study the effect of a single, double, and electron transport layer sandwiched Poly(4-vinylpyridine) (PVPy here on) on the charge injection balance and on the overall device performance of InP-based red quantum dot light emitting diodes (red QD-LEDs). The results showed general improvement of device characteristic performance metrics such as operational life with incorporation of a PVPy interlayer. The best performance was observed at a lower concentration of PVPy (@ 0.1 mg/mL) in interlayer with continual worsening in performance as PVPy concentration in the interlayer increased in other fabricated devices. The AFM images obtained for the different materials reported improved surface morphology and overall improved surface properties, but decreased overall device performance as PVPy concentration in interlayer was increased. Furthermore, we fabricated two special devices: in the first special device, a single 0.1 mg/mL PVPy sandwiched between two ZnO ETL layers, and in the second special device, two 0.1 mg/mL PVPy interlayers were inter-sandwiched between two ZnO ETL layers. Particular emphasis was placed on monitoring the maximum obtained EQE and the maximum obtained luminance of all the devices. The first special device showed better all-round improved performance than the second special device compared to the reference device (without PVPy) and the device with a single 0.1 mg/mL PVPy interlayer stacked between ZnO ETL and the emissive layer.

## 1. Introduction

Quantum light-emitting diodes (QD-LEDs) have emerged as next generation devices owing to their superior color purity, high material stability, and low fabrication cost, with possible fields of application including high-brightness displays, wearable/flexible displays, flat-panel displays, automotive displays, transparent displays, augmented/virtual reality displays, and solid state lighting [1,2,3]. The emission mechanism of QD-LEDs is a result of a radiative (balanced charge) recombination between positive (holes) and negative (electrons) charge carriers forming excitons, which produce light inside an emission layer [4,5,6]. The charge carriers are injected into the emission layer from the anode and the cathode from the adjacent hole and electron injection and transport layers [7,8]. As of today, plenty of research has utilized organic-inorganic hybrid QD-LED device structures consisting of an n-type metal oxide as the electron transport layer (ETL) material and a p-type small molecule polymer as the hole transport layer (HTL) material [9,10,11]. Of all the previously studied electron transport layer materials, Zinc Oxide nanoparticles (ZnO NPs) still remain the most widely studied, and they are used as an electron transport layer (ETL) material due to their high ability to inject electrons into the emissive layer––an ability that stems from their high transparency and their high electron mobility [12,13]. Typical hole transport materials (HTMs) currently used in QD-LED fabrication have low hole mobility values, with orders between 10^−3^ and 10^−4^ cm^2^V^−1^S^−1^ [14,15]. Tris(4-cabazoyl-9-ylphenyl)amine (TCTA) utilized in our studies has a hole mobility in this range [16].

Despite the advancements in QD-LED device performance owing to prior extensive research efforts, there are still a few issues to resolve in order to realize large-scale production. The most critical issue is imbalanced charge injection into the quantum dot emissive layer during the operation of the device [17]. Along with this, a multitude of other phenomena that negatively affect device performance, such as the formation of leakage current at the interface between the emissive layer and metal oxide electron transport material [18], negative charging of the emissive layer by excessive electrons in the metal oxide ETL [19], and the intrinsic difference of the charge carrier density and charge mobility between inorganic ETLs and organic HTLs [19,20], are all issues related to the electron-hole injection imbalance into the emissive layer. Some of the techniques currently utilized in the optimization of electron injection into the emissive layer include the use of various thicknesses for electron layer materials [7], inserting a thin interlayer layer between the emissive layer and the metal oxide electron transport material [21], and the doping of the electron transport layer for effective charge transportation [22].

Of all the abovementioned methods, we decided to adopt inserting a thin interlayer layer between the emissive layer and the metal oxide. This was undertaken in order to reduce the rate of electron injection from the cathode through to within the emissive layer, which, in turn, establishes a balanced rate of exciton formation within the emissive layer [17]. To achieve charge balance injection through insertion of an insulating interlayer, it is important to study the insulating material that will be used. Recently, a non-conjugate polymer––namely, Poly(4-vinylpyridine)––has been used as an insulating layer to reduce the rate of electron injection, and it has garnered considerable success due to its ability to form a strong interaction with the ZnO-based electron transport layer [23,24]. In this paper, we studied the effect of a single, double, and electron transport layer sandwiched PVPy on the overall performance of InP-based red quantum dot light-emitting diodes (QD-LEDs).

From these results, we obtained valuable insight into the importance of establishing charge injection balance between the electron transport layer and the emissive layer in QD-LEDs, which further highlights its importance to regulating electron injection into the emissive layer.

## 2. Materials and Experiments

### 2.1. Materials

Indium Tin Oxide coated glass substrates (ITO glass) were used in all of the fabricated devices. ZnO nanoparticles solution was used to make all the electron transport layers for the three devices we investigated. [25,26]. Zinc acetate dehydrate (Zn(CH_3_COO)_2_.2H_2_O) ≥ 98%, Poly(4-vinylpyridine) (PVPy), Isopropyl alcohol(2-propanol), and Tris(4-cabazoyl-9-ylphenyl)amine were purchased from Sigma Aldrich. Molybdenum Oxide (MoO_3_) was purchased from OSM. The silver used as the cathode was purchased from TCI.

### 2.2. Preparation of PVPy Solutions for Stacked Interlayer

The respective masses of PVPy (0.1, 0.2, 0.3 and 0.5 mg) were weighed, and each mass was dissolved into a 1 mL volume of Isopropyl alcohol (2-propanol).

### 2.3. Device Fabrication

Before fabrication, glass substrates coated with indium tin oxide (ITO) were cleaned first through ultra-sonication with acetone and then by cleaning with Isopropyl alcohol for 10 min, respectively, and they were thereafter dried inside an oven at 100 °C for 2 h. The pre-cleaned ITO glass substrates were then treated with UV-ozone inside an UV cleaner for 15 min, and were then transferred into a N_2_-filled glass box for the experiment. The reference device (without PVPy) was fabricated according to the inverted structure: ITO/ZnO NPs (20 nm)/InP-based red QDs (20 nm)/TCTA (50 nm)/MoO_3_ (10 nm)/Ag (80 nm). All of the devices into which the PVPy was incorporated were fabricated according to the inverted structure: ITO/ZnO NPs (20 nm)/PVPy (different conc.)/InP-based red QDs (20 nm)/TCTA (50 nm)/MoO_3_ (10 nm)/Ag (80 nm). Inside an N_2_ gas-filled glove box, ZnO nanoparticles solution was filtered first and then deposited through a syringe onto the pre-cleaned ITO substrate. Spin coating was carried out at 2000 rpm for 60 s, which was then followed by annealing at 90 °C for 30 min. The different PVPy solutions that formed the PVPy interlayer were spin coated at 3500 rpm for 45 s onto the substrate pre-coated with ZnO ETL, and were then annealed at 100 °C for 10 min. The emissive layer (InP-based red QDs) was spin coated onto the substrate at 3500 rpm for 60 s, and this was then followed by annealing at 120 °C for 30 min. The TCTA, MoO_3_, and Ag electrode used were then deposited through a thermal evaporation process under high vacuum conditions.

**Figure 1:**
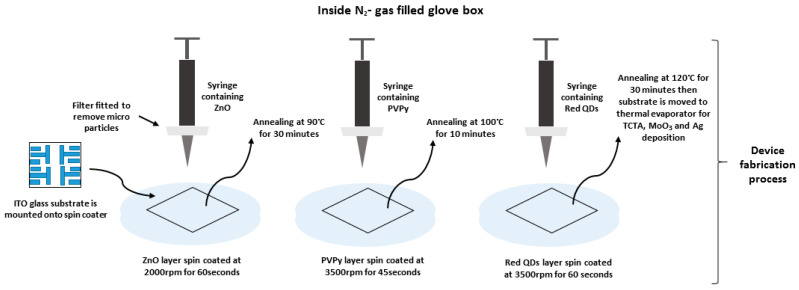
Shows a schematic diagram of the device fabrication process used in the experiment.

In addition, two special devices were fabricated. The first special device utilized a single 0.1 mg/mL PVPy interlayer sandwiched between double ZnO ETL layers with a device structure as follows: ITO/ZnO ETL (20 nm)/0.1 mg/mL PVPy/ ZnO ETL (20 nm)/InP based red QDs (20 nm)/TCTA (50 nm)/MoO_3_ (10 nm)/Ag (80 nm). The other special device was fabricated with two 0.1 mg/mL PVPy layers and two ZnO ETL layers, with an inverted device structure as follows: ITO/ZnO ETL (20 nm)/0.1 mg/mL PVPy/ZnO ETL (20 nm)/0.1 mg/mL PVPy/InP based red QDs (20 nm)/TCTA (50 nm)/ MoO_3_ (10 nm)/Ag (80 nm). As performed previously, all PVPy layers in these particular devices were spin coated onto the substrate at 3500 rpm for 45 s and annealed at 100 °C for 10 min. All ZnO ETL layers were also spin coated onto the substrate at 3500 rpm for 60 s, and were then annealed at 90 °C for 30 min. All non-solution layers followed a similar deposition procedure, as previously mentioned. The device fabrication process is clearly displayed in the flow diagram in Figure 1.

### 2.4. Characterization

The absorption spectra of the different PVPy concentrations were measured using UV-visible absorption spectroscopy (UV-2600i), and the resulting data were plotted to obtain the absorption region of the respective concentrations. The current voltage (*I-V*) and luminance voltage (*L-V*) characteristic data of the devices were obtained under ambient conditions using a computer-controlled Keithely 6487 Source meter with sequential voltage increments of 0.5 volts. The electroluminescence (EL) spectra, which yielded luminance (cd/m^2^), external quantum efficiency (EQE %), current density (mA/cm^2^), power efficiency (Im/W), current efficiency (cd/A), and spectral intensity data, were measured with a scanning spectroradiometer (PR-655). The surface morphology and the root mean square value of surface roughness (RMS roughness) was determined through Atomic Force Microscopy imaging.

## 3. Results and Discussion

**Figure 2:**
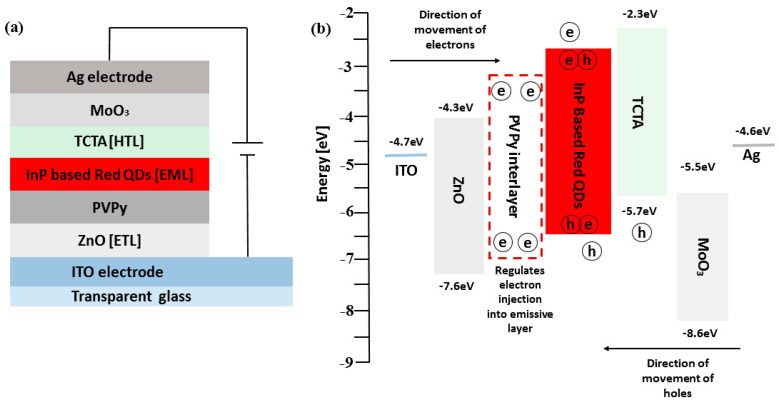
(**a**) The schematic diagram of the inverted layered devices incorporating a PVPy interlayer. (**b**) Shows the energy band diagram of the co-responding device structure of the devices with their respective band energies.

**Figure 3:**
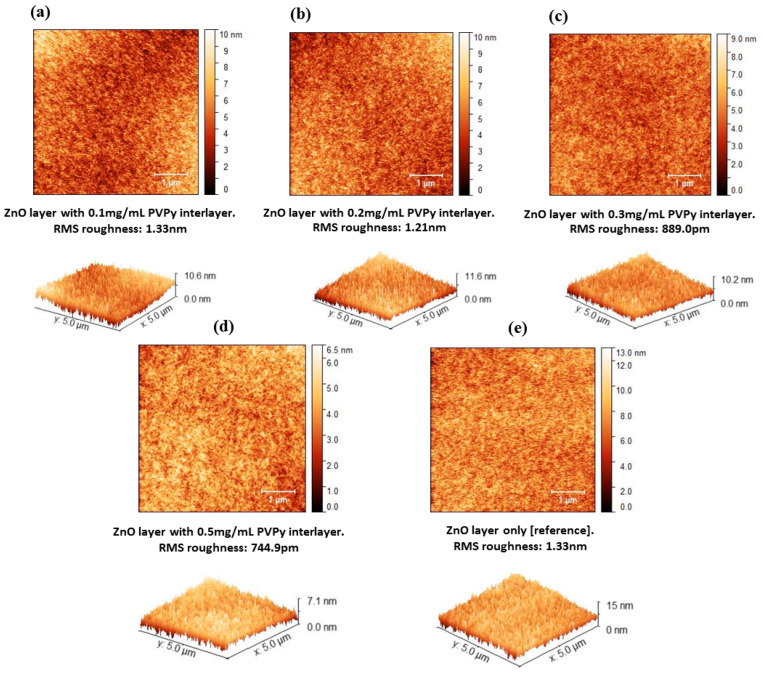
AFM images of the ZnO/PVPy (different concentrations) thin films for surface roughness comparison. (**a**) 0.1mg/mL PVPy (**b**) 0.2mg/mL PVPy (**c**) 0.3mg/mL PVPy (**d**) 0.5mg/mL PVPy (**e**) only ZnO thin film without a PVPy.

The schematic device structure of the InP-based red QDs layered inverted QD-LED devices is shown in Figure 2a, which consists of ITO/ZnO (ETL) (20 nm)/PVPy (different conc.)/InP based red QDs (20 nm)/TCTA (HTL) (50 nm)/MoO_3_ (HIL) (10 nm)/Ag (80 nm). The energy band diagram of the layered QD-LED devices is further shown in Figure 1b, with each layer mapped to its corresponding energy band energies. The work function of ITO and Ag is −4.7 eV and −4.6 eV, respectively [1]. The HOMO level of ZnO is −7.6 eV and its LUMO is −4.3 eV [27]. For TCTA, the HOMO level is −5.7 eV and LUMO is −2.3 eV [28]. For MoO_3_, HUMO level is −8.6 eV and LUMO level −5.5 eV [29]. The energy band diagram for the reference device was used as the template for all of the other devices fabricated during the experiment. The reference device was fabricated using a ZnO electron transport layer without a PVPy interlayer, and its corresponding device performance characteristics, including EQE and luminance measurements, were obtained. Throughout the experiment, the variation of the maximum EQE and the maximum luminance with subsequent incorporation of the PVPy interlayer were the main parameters that we focused on. In the initial stages of the experiment, we measured the root mean square roughness of ZnO NPs + PVPy by spin coating onto clean bare glass—first, ZnO NPs at 2000 rpm for 60 s, and then annealing them for 30 min at 90 °C—followed by spin coating the different PVPy concentrations on top of ZnO NPs pre-coated glass at 3500 rpm and then annealing them at 100 °C. The coated glass substrates were taken for the AFM imaging, which produced the results in Figure 3.

Figure 3a–d shows AFM images of the different solutions of PVPy deposited on bare glass for surface roughness comparison. The reference (ZnO ETL only in Figure 3e) was recorded to have the highest root mean square (RMS) roughness of the surface for all of the tested materials. Despite the similarity between the RMS roughness values of ZnO only and ZnO with PVPy 0.1 mg/mL, the RMS roughness values of all materials here decreased with the increasing concentration of the PVPy layer, which implies an increase in the surface smoothness and the establishment of better surface properties [10]. (The RMS roughness values for ZnO only (reference device) were: 1.33 nm, with 0.1 mg/mL: 1.33 nm, with 0.2 mg/mL: 1.21 nm, with 0.3 mg/mL: 889.0 pm, with 0.5 mg/mL: 744.9 pm).

**Figure 4:**
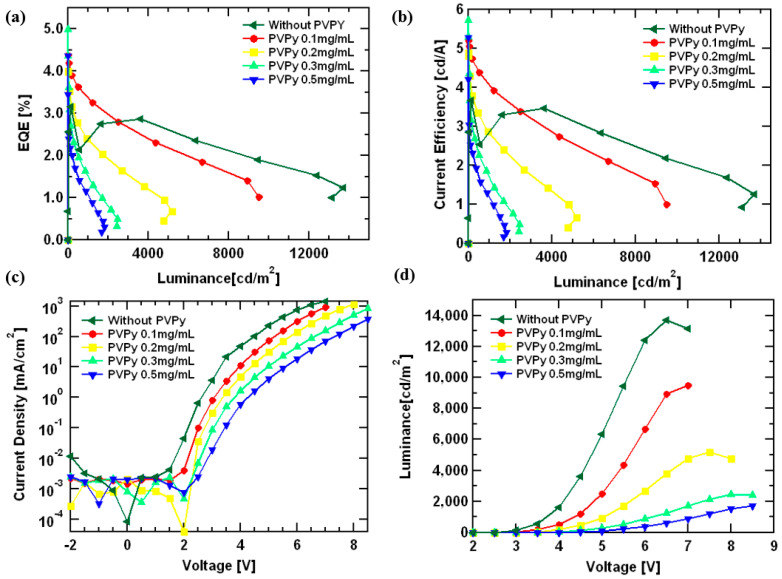
(**a**) A plot of the External Quantum Efficiency (EQE) versus luminance. (**b**) Current Efficiency versus luminance. (**c**) Current Density versus Voltage. (**d**) Luminance versus Voltage. All of the diagrams in Figure 4 explain the device fabricated using different concentrations of PVPy.

Figure 4 shows the *J-V-L* measurements for all of the devices containing a PVPy interlayer compared to the results from the reference device (without PVPy). Figure 4a shows the variation of external quantum efficiency (EQE) with luminance. The reference device (without PVPy) registered the highest maximum luminance values (@ 13,700 cdm^−2^) compared to devices with a PVPy interlayer. For the devices with a PVPy interlayer, the maximum luminance decreased as the concentration of PVPy in the interlayer increased (with 0.1 mg/mL: 9501 cdm^−2^; with 0.2 mg/mL: 5173 cdm^−2^; with 0.3 mg/mL: 2465 cdm^−2^; and with 0.5 mg/mL 1824 cdm^−2^). The maximum EQE values for all devices with a PVPy interlayer were registered at the turn-on voltage, which makes the EQE@100 nits an important parameter for these particular devices. In that regard, Table 1 shows the values of EQE@100 nits and their corresponding maximum observed luminance values to further explain the prior findings. Figure 4b shows current efficiency versus luminance, and the device showed a reduction in efficiencies as the PVPy concentration increased. This reduction in efficiencies with a higher concentration can be attributed to an imbalanced electron injection into the emissive layer [30]. Figure 4c shows a current density versus voltage plot of the devices, showing the current density to slightly decrease for the devices containing the PVPy interlayer. This illustrates the insulating nature of the PVPy interlayer. The turn-on voltage for the different devices ranges between 2.5 V and 3.5 V, indicating a relative reduction in the turn-on voltage of the PVPy-based devices. The results show that the introduction of a PVPy interlayer into the devices, regardless of the concentration, has little to no effect on hole injection. The reduction of the current density can thus be attributed to the suppression of the leakage current from the emissive layer to the ZnO layer and low conductivity of the PVPy interlayer. Maximum observed luminance values in the reference device were higher than in the devices containing the PVPy interlayer. The observed maximum luminance values decreased as the concentration of the PVPy in the PVPy interlayer increased. This was a result of the formation of quenching sites or trap states on the surface of the ZnO NPs electron transport layer, which were possibly created as a result of the presence of the PVPy [31]. Figure 4d shows luminance versus voltage. All of the devices had a turn-on voltage between 2.5 V and 3.5 V, and they could be operated to voltages upwards of 8 V. The rate of the injection of electrons was faster than the rate of injection of the hole injection into the emissive layer. [32] In addition, the rates of the charge carrier injection increase with the increasing applied voltage [33]. A steady increase in the luminance is observed initially at lower voltages due to the low amount of charge carriers injected into the devices in this region. For the reference device (without PVPy), the high electron mobility of ZnO ETL in contact with the emissive layer facilitates a faster charge carrier injection into the emissive layer. On the other side, TCTA has a low charge carrier mobility, and, thus, the charge carrier injection is slower [16]. This creates an imbalance due to the presence of a higher amount of electrons than holes inside the emissive for radiative recombination as the applied voltage is increased. In the device without PVPy, all of the available holes participate in the radiative recombination, leaving un-recombined electrons inside the emissive layer. After the available holes participate in exciton formation coupled with the continuous injection of electrons at high voltages into the device, a surplus buildup of excess electrons inside the emissive layer occurs, and with no holes for radiative recombination, the device dies off quickly. In the case of the device with PVPy, the insulating nature of PVPy decreases the amount of electrons that enter into the emissive layer, which establishes a charge carrier injection balance and reduces the rate of electron surplus build-up inside the emissive layer, even at higher voltages. This leads to an increase in the device’s operational time, hence the shift in position where the maximum luminance is observed.

**Figure 5:**
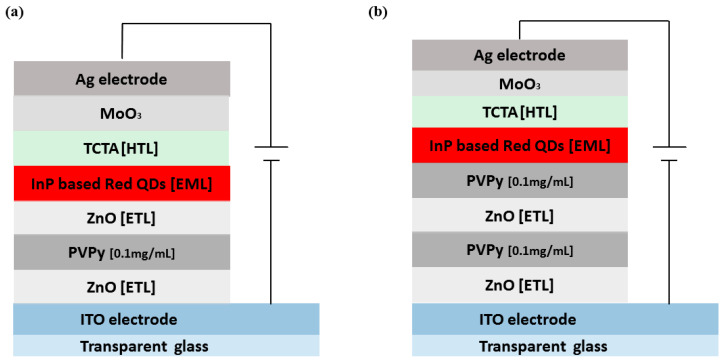
(**a**) The structure of a device fabricated with two ZnO electron transport layers and a single PVPy 0.1 mg/mL concentration interlayer. (**b**) The structure of a device fabricated with two ZnO electron transport layers and a double PVPy 0.1 mg/mL concentration interlayer. All devices utilized the inverted structural framework.

**Figure 6:**
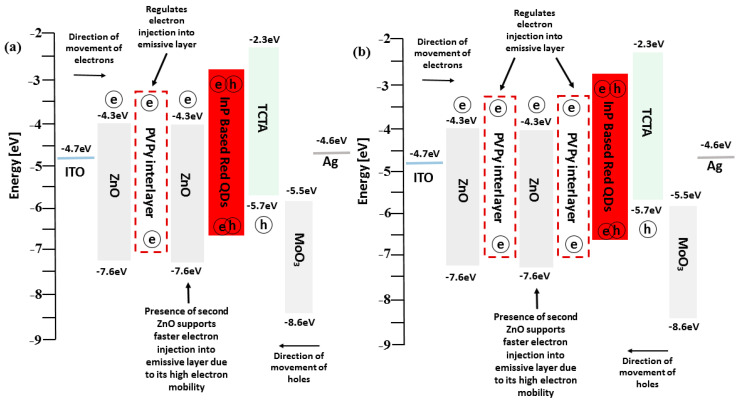
(**a**) Energy level diagram of the first special device (a single 0.1 mg/mL PVPy layer with double ZnO NPs layers). (**b**) Energy diagram of second special device (two 0.1 mg/mL PVPy interlayers with double ZnO layers).

In addition to the devices using a single PVPy interlayer, we prepared two special devices. The first device utilized a 0.1 mg/mL PVPy layer sandwiched between two ZnO ETL layers—one connected to the ITO electrode and the other connected to the emissive layer, as shown in Figure 5a. The other device used two 0.1 mg/mL PVPy interlayers and two ZnO ETL layers, as shown in Figure 5b. All of the corresponding energy diagrams are shown in Figure 6. These devices were tested to determine their performance characteristics and how they compare to both the reference device (the device without a PVPy interlayer) and the device with a single 0.1 mg/mL PVPy interlayer.

**Figure 7:**
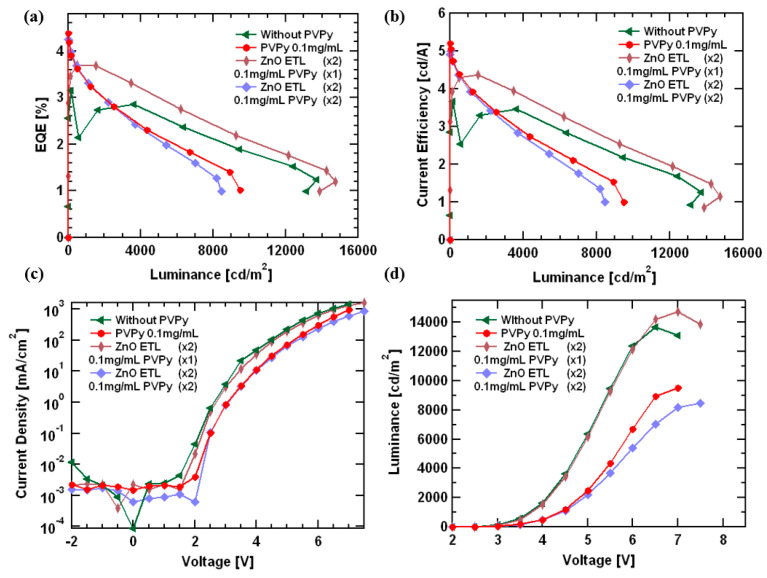
(**a**) A plot of the External Quantum Efficiency (EQE) versus luminance. (**b**) Current Efficiency versus luminance. (**c**) Current Density versus Voltage. (**d**) Luminance versus Voltage. All of the diagrams in Figure 7 explain devices fabricated using 0.1 mg/mL PVPy interlayer.

We noticed an improvement in device performance metrics. The first special device featured in Figure 5a showed higher maximum observed luminance values of 14,750 cdm^−2^ and also higher EQE@100 nits values of 3.36% compared to the reference device (without PVPy luminance @ 13,700 cdm^−2^, EQE@100 nits 2.96%). The second special device featured in Figure 5b registered lower maximum observed luminance values than the reference device (@ 8446 cdm^−2^) with a higher EQE@100 nits of 4.11% than the reference device (device without PVPy), as shown by the EQE-Luminance (in Figure 7a) and the Luminance-Voltage plots (in Figure 7d), respectively. These results show that the use of the sequential ZnO-PVPy-ZnO configuration could be the possible reason for the improvement in the device performance metrics, thereby reporting a better carrier injection balance than in the device that uses a single PVPy interlayer from both special devices. For the first special device, due to the high electron mobility of ZnO, the presence of a second ZnO ETL layer in contact with the emissive layer facilitates faster electron injection into the emissive layer, which, in turn, facilitates improved recombination and exciton formation inside the emissive layer. However, for the second special device, the subsidized improvement of the device performance metrics compared to the first special device can be attributed to the presence of an extra PVPy layer in contact with the emissive layer, which further blocks more electrons from entering the emissive layer. This reduced rate of electron injection causes the rate of radiative recombination and the exciton formation inside the emissive layer.

To further understand how electron transport is affected in the different devices, we fabricated electron-only devices (EOD). The device configurations were as follows:

i. ITO/ZnO/nP-red QDs/ZnO/Ag [reference] (without PVPy).
ii. ITO/ZnO/PVPy [0.1 mg/mL]/InP-red QDs/ZnO/Ag.
iii. ITO/ZnO/PVPy [0.2 mg/mL]/InP-red QDs/ZnO/Ag.
iv. ITO/ZnO/PVPy [0.3 mg/mL]/InP-red QDs/ZnO/Ag.
v. ITO/ZnO/PVPy [0.5 mg/mL]/InP-red QDs/ZnO/Ag.
vi. ITO/ZnO/PVPy [0.1 mg/mL]/ZnO/InP-red QDs/ZnO/Ag.
vii. ITO/ZnO/PVPy [0.1 mg/mL]/ZnO/PVPy [0.1 mg/mL]/InP-red QDs/ZnO/Ag.

**Figure 8:**
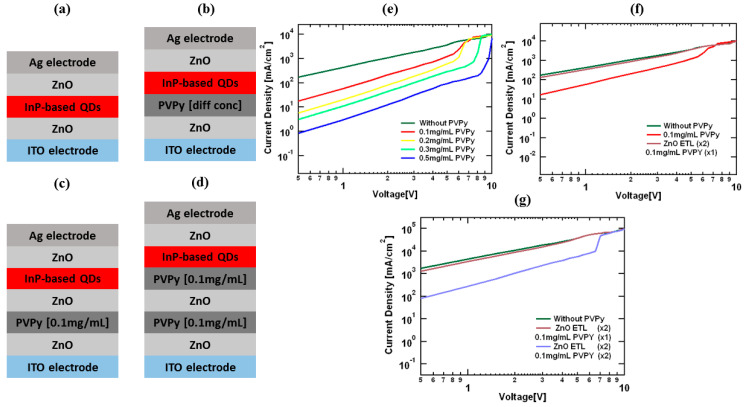
(**a**) The configuration of the reference EOD device. (**b**) The configuration of EOD devices with PVPy. (**c**) The EOD device configuration for the first special device (single 0.1 mg/mL PVPy interlayer with double ZnO ETL layers). (**d**) EOD device configuration of the second special device (double 0.1 mg/mL PVPy interlayers with double ZnO ETL layers). (**e**–**g**) Current density versus voltage graphs for the different configurations.

In Figure 8e, current density variation of the reference electron device (without a PVPy interlayer) is compared to the electron-only devices fabricated with different concentrations of PVPy in the incorporated PVPy interlayer. The current density for the various devices is seen to decrease as the concentration of PVPy in the PVPy interlayer is increased. The continual decrease in the current density with increasing PVPy concentration implies a decrease in the rate of electron injection, since the PVPy layer blocks more movement of electrons in the red QDs layer [32]. Figure 8e shows a side-by-side comparison of current density variation in the reference device with a single PVPy interlayer and Figure 8f shows current density variation in the first special device (single 0.1 mg/mL PVPy interlayer with double ZnO ETL layers). The trend for the first special device EOD configuration in Figure 8f is closely similar to that of the reference device, with a slight decrease in the current density. This observation points to a steady injection of electron into the red QDs layer, owing to the high electron mobility of the second ZnO layer in the structural configuration of ZnO-PVPy-ZnO. Figure 8g shows the reference device with two special devices. The second special device is seen to suffer a greater decrease in current density than the first special device. This is brought about by the presence of an extra PVPy layer, which hinders more electron injection. This implies that the first special device’s EOD configuration is more stable.

**Figure 9:**
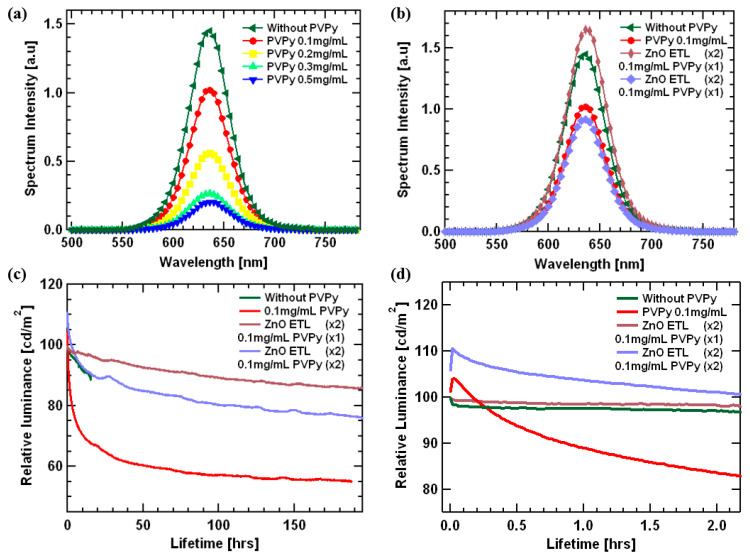
(**a**,**b**) The spectrum intensity versus wavelength. These confirm formation of the excitons within the InP red Quantum Dot (QDs) emissive layer. (**c**) A plot of Relative luminance versus lifetime. (**d**) An expanded lifetime graph between 0.0 h and 2.0 h.

Figure 9a,b shows the spectrum intensity of the light emitted from the emissive layer plotted against wavelength. All devices displayed red characteristic of InP-based red quantum dots ranging between 550 nm and 700 nm, and the characteristic profiles were similar for all devices despite the insertion of a PVPy interlayer. This further demonstrates that the exciton formation zone was not affected by the introduction of the PVPy interlayer, thus reporting the formation of the excitons inside the emissive layer [34].

Figure 9c shows relative luminance versus device lifetime. The testing was conducted for all devices, with the starting luminance set at 100 nits. The trends show that the relative luminance decreases faster for the reference device (without PVPy) compared to the devices containing a PVPy interlayer. In regards to the lifetime of the device, the time taken for the relative luminance to decrease to 90% of the original value (namely, T_90_) was measured and put into consideration as one of the metrics for determining the performance of the device. The device consisting of a single 0.1 mg/mL PVPy interlayer with double ZnO ETL had T_90_ equal to 87.3 h, which shows improved stability in comparison to all of the other fabricated devices. (Reference device T_90_ = 14.4 h, 0.1 mg.mL PVPy T_90_ = 0.86 h and double PVPy with double ZnO layer T_90_ = 16.8 h). With these results, we can see that the device fabricated using single PVPy with double ZnO has better overall performance statistics in comparison to all of the other prior fabricated devices. This would thereby prompt the idea that the more PVPy interlayers, the better the charge injection balance.

## 4. Conclusions

In summary, we studied the effect of a single, double, and ETL sandwiched Poly(4-vinylpyridine) (PVPy) interlayer on the maximum observed external quantum efficiency (EQE), the maximum observed luminance, and, thereby, the overall performance of InP-based red quantum dot light-emitting diodes (red QD-LEDs). The results obtained from AFM imaging suggest that in cooperation with a PVPy interlayer on top of a ZnO layer, the surface morphology is improved, which, in turn, improves the surface properties of the ZnO layer with increasing PVPy concentrations. However, though the surface properties improved, the overall device performance metrics were negatively affected by the presence of high PVPy concentration devices registering lower maximum observed luminance values with higher EQE values. It was determined initially that the device with a 0.1 mg/mL PVPy concentration shows device performance metrics that tended towards those of the reference device (without a PVPy interlayer), which compares to all of the other devices with a PVPy interlayer. Furthermore, the addition of a PVPy interlayer had little to no effect on the turn-on voltage of the devices, with devices fabricated with a PVPy interlayer having a turn-on voltage between 2.5 V and 3.0 V, which is relatively similar to the reference device (without a PVPy interlayer).

Two special devices were fabricated to determine the effect of using multiple layers on the overall performance of the QD-LEDs. The first special device had a single 0.1 mg/mL PVPy interlayer with double ZnO ETL layers, and the other special device had two 0.1 mg/mL PVPy interlayers with double ZnO ETL layers. The first special device scored a maximum observed luminance of 14,750 cdm^−2^ and a higher EQE@100 nits value of 3.36% compared to the reference device. The second special device had a lower maximum observed luminance of 8446 cdm^−2^ than the reference device but higher EQE@100 nits of 4.11%. Both special devices achieved a longer operational time compared to the reference device. The first special device had T_90_ equal to 87.3 h, while the second special device had T_90_ equal to 16.8 h, and these are both better results compared to the reference device (T_90_ = 14.4 h). The results obtained from the first special device were attributed to the high electron mobility of the second ZnO layer in contact with the emissive layer, which accelerates the injection of electrons into the emissive layer. For the second special device, the presence of a second PVPy interlayer in contact with the emissive layer further blocks more electrons from entering the emissive layer, thereby causing a reduction in the rate of electron injection, which, in turn, reduces the rate of radiative recombination inside the emissive layer. In conclusion, we can see that the device fabricated using the ZnO-PVPy-ZnO configuration plays a vital role in facilitating the balance of electron injection into the emissive layer.

## Figures and Tables

**Table 1:** Important device metrics for all of the devices fabricated for the experiment.

Device	Turn-On Voltage [V]	Max EQE [%]	EQE@100 nits [%]	Current Efficiency@100 nits [cd/A]	Device Max Luminance [cd/m^2^]	Lifetime @ T_90_
Reference device (Without PVPy)	2.5	3.15@3.0 V	2.96	3.47	13,700	14.4
0.1 mg/mL PVPy	2.5	4.39@2.5 V	4.06	4.91	9501	0.86
0.2 mg/mL PVPy	2.5	4.01@3.0 V	3.15	4.15	5173	-
0.3 mg/mL PVPy	2.5	4.99@2.5 V	2.92	3.15	2465	-
0.5 mg/mL PVPy	2.5	4.36@3.0 V	2.17	2.54	1825	-
Double ZnO ETL Single 0.1 mg/mL PVPy	2.5	3.69@3.5 V	3.36	3.78	14,750	87.3
Double ZnO ETL Double 0.1 mg/mL PVPy	2.5	4.25@2.5 V	4.11	4.85	8446	16.8

## Data Availability

Data will be made available on request.
